# Phospho-mTOR in non-tumour and tumour bladder urothelium: Pattern of expression and impact on urothelial bladder cancer patients

**DOI:** 10.3892/ol.2014.2392

**Published:** 2014-07-30

**Authors:** JULIETA AFONSO, ADHEMAR LONGATTO-FILHO, VITOR MOREIRA DA SILVA, TERESINA AMARO, LÚCIO L. SANTOS

**Affiliations:** 1 Life and Health Sciences Research Institute (ICVS), School of Healh Sciences (ECS) University of Minho, Braga 4710-057, Portugal; 2 ICVS/3B’s, PT Government Associate Laboratory, Braga 4710-057/Guimarães 4806-909, Portugal; 3 Laboratory of Medical Investigation (LIM 14), Faculty of Medicine, São Paulo State University, São Paulo 01246-000, Brazil; 4 Molecular Oncology Research Center, Barretos Cancer Hospital, Barretos, São Paulo 14784-400, Brazil; 5 Department of Urology, Portuguese Institute of Oncology (IPO), Porto 4200-072, Portugal; 6 Experimental Pathology and Therapeutics Research Center, Portuguese Institute of Oncology (IPO), Porto 4200-072, Portugal; 7 Department of Surgical Oncology, Portuguese Institute of Oncology (IPO), Porto 4200-072, Portugal; 8 Faculty of Health Sciences, University Fernando Pessoa, Porto 4200-150, Portugal

**Keywords:** p-mTOR, urothelial bladder cancer, pattern of expression, umbrella cells

## Abstract

Urothelial bladder carcinoma (UBC) is heterogeneous in its pathology and clinical behaviour. Evaluation of prognostic and predictive biomarkers is necessary, in order to produce personalised treatment options. The present study used immunohistochemistry to evaluate UBC sections containing tumour and non-tumour areas from 76 patients, for the detection of p-mTOR, CD31 and D2-40 (blood and lymphatic vessels identification, respectively). Of the non-tumour and tumour sections, 36 and 20% were scored positive for p-mTOR expression, respectively. Immunoexpression was observed in umbrella cells from non-tumour urothelium, in all cell layers from non-muscle-invasive (NMI) tumours (including expression in superficial cells), and in spots of cells from muscle-invasive (MI) tumours. Positive expression decreased from non-tumour to tumour urothelium, and from pT1/pTis to pT3/pT4 tumours; however, the few pT3/pT4 positive cases had worse survival rates, with 5-year disease-free survival being significantly lower. Angiogenesis occurrence was impaired in pT3/pT4 tumours that did not express p-mTOR. In conclusion, p-mTOR expression in non-tumour umbrella cells is likely a reflection of their metabolic plasticity, and extension to the inner layers of the urothelium in NMI tumours is consistent with an enhanced malignant potential. The expression in cell spots in a few MI tumours and absence of expression in the remaining tumours is intriguing and requires further research. Additional studies regarding the up- and downstream effectors of the mTOR pathway should be conducted.

## Introduction

Bladder cancer is the second most common urological malignancy and represents a significant epidemiological problem. There was an estimated 386,300 new cases and 150,200 deaths in 2008, worldwide (1). Urothelial carcinoma is the most common histological subtype of bladder cancer in developed countries (2). The majority of patients present with non-muscle-invasive (NMI) tumours that, although lack aggressive histopathological features, frequently recur, which therefore demands for long-term follow-up and repeated intervention. High-grade NMI lesions harbor an enhanced risk of progression to muscle-invasive (MI) disease. MI tumours have significant metastatic potential (3), for which radical cystectomy (RC) with bilateral pelvic and iliac lymphadenectomy is the most common treatment (4,5). This surgical approach provides a cure for the majority of patients with organ-confined lesions (6). However, regional lymph node and visceral metastasis are common findings, advocating the association of neoadjuvant and adjuvant therapies. Cisplatin-containing combinations are standard treatment options of care for patients with urothelial bladder carcinoma (UBC), but heterogeneity in the response to the treatment and patient fragility significantly impairs the survival benefits (7). Up to 50% of patients with MI-UBC will eventually succumb to metastatic disease (6).

Current investigations have started to research the molecular pathogenesis of bladder tumours, in an attempt to identify biomarkers of aggressiveness, response to chemotherapy and potential therapeutic targets. The mammalian target of rapamycin (mTOR) intracellular pathway represents a potential target. mTOR belongs to the phosphoinositide-3-kinase (PI3K)-related kinase family, and is centrally involved in the transduction of proliferative factors induced by the PI3K/Akt signalling pathway, to the level of mRNA and ribosomes (8–11). The *mTOR* gene encodes a protein product that functions as a component of two complexes, mTOR complex 1 (mTORC1) and mTORC2 (10). Through its interactions with regulatory-associated protein of mTOR, contained in mTORC1, and rapamycin-insensitive companion of mTOR, contained in mTORC2, activated mTOR regulates protein translation, cell cycle progression, actin cytoskeleton organization, cell migration and survival (8–11). Furthermore, mTOR signalling can mediate angiogenesis and lymphangiogenesis by increasing vascular endothelial growth factor secretion. In addition, mTOR functions in the crosstalk between tumour and endothelial cells (12–14). Increased mTOR activity, as well as increased levels of phosphorylation of its downstream targets, eukaryotic translation initiation factor 4E binding protein 1 and ribosomal protein S6 kinase, have been detected in a significant percentage of human tumours (15–22). Rapamycin (sirolimus) and rapamycin analogues (such as temsirolimus and everolimus), selectively inhibit the mTOR pathway, and have demonstrated potent antitumour effects both *in vitro* and *in vivo* (23–26). A number of these compounds have already obtained Food and Drug Administration approval for the treatment of human malignancies (27), and numerous clinical trials are ongoing (28,29), including trials for patients with UBC (30). There has been little research into the levels of mTOR activation in bladder tumour tissue, with inconsistencies in the existing results. Hansel *et al* (31) reported the expression of phosphorylated mTOR (p-mTOR) in 74% (90/121) of MI UBCs, and a significant association with increased pathological stage and reduced disease-specific survival was noted. Conversely, Makhlin *et al* (32) found that p-mTOR expression was increased in malignant versus normal urothelium in only 32% (65/203) of tumours, and no association with clinicopathological and outcome parameters was observed (32).

The present study aimed to evaluate the pattern of expression and the clinical and prognostic significance of p-mTOR, by immunohistochemistry, in 76 patients with high risk of UBC progression. Angiogenesis and lymphangiogenesis occurrence was also evaluated by immunohistochemistry, in order to correlate blood vessel density (BVD) and lymphatic vessel density (LVD) with p-mTOR expression.

## Methods

### Patient and tumour samples

The records from patients who were clinically diagnosed with a high risk of progressive UBC (high-grade NMI and MI tumours) and treated by RC and limited lymphadenectomy at the Portuguese Institute of Oncology (IPO) (Porto, Portugal), from January 1996 to December 2005, were retrospectively reviewed. Prior approval was obtained from the ethics committee of the Portuguese Institute of Oncology, and written informed consent was obtained from the patient/the patient’s family. During this period, 223 RCs were performed. Exclusion criteria included the diagnosis of urothelial carcinomas with variant histology, squamous cell or adenocarcinomas, prior radiation, neoadjuvant or adjuvant chemotherapy treatments, insufficient follow-up time and/or tumour samples inadequate for further study (such as samples without adjacent non-tumour urothelium). A final cohort of 76 patients were eligible for the study. Each cystectomy specimen was examined following the guidelines of the College of American Pathologists (33). Two independent pathologists (A.L-F. and T.A.) reviewed haematoxylin-eosin-stained sections according to the standard histopathological examination methods, considering the American Joint Committee on Cancer (34) and the World Health Organization-WHO (WHO 1999 and WHO 2004) (35,36) classification systems. Table I summarises the clinicopathological parameters.

The 61 patients with MI tumours (80%) had RC as their first treatment, while the 15 patients with NMI tumours (20%) had a previous therapeutic transurethral resection and BCG instillation. When disease recurrence occurred, or when multiple carcinoma *in situ* (CIS) lesions were observed in the surgical specimen, these patients were then treated by RC. The mean and median follow-up times were 35 and 20 months (range, 1–132 months), respectively. Recurrence, disease-free survival (DFS) and overall survival (OS) rates were defined as the reappearance of UBC (loco-regional metastasis or distant metastasis) >3 months following RC, the time from RC until recurrence, and the time from RC until the patient succumbed to the cancer or the last clinical assessment, respectively.

### Immunohistochemistry and evaluation of staining

Immunohistochemical staining to detect p-mTOR was performed on paraffin-embedded 4 μm UBC tissue sections following the horseradish peroxidase two-step peroxidase conjugated polymer method (EnVision™+ system; DakoCytomation, Glostrup, Denmark), according to the manufacturer’s instructions. The monoclonal rabbit anti-human phospho-mTOR primary antibody (Ser2448; Cell Signalling Technology, Inc., Danvers, MA, USA) was used in a 1:500 dilution and incubated on the sections overnight at 4°C. Samples processed by omitting the primary antibody were used as negative controls. A breast tumour with known immunoreactivity for p-mTOR was used as a positive control. Blood and lymphatic endothelial cells were immunohistochemically stained by monoclonal mouse anti-human CD31 and D2-40 DakoCytomation antibodies (DakoCytomation), as previously described (37).

The immunostained sections were examined by light microscopy (Motic BA310 Series; Motic Spain, S.L.U., Barcelona, Spain) by two independent observers (A.L-F. and T.A.) who had no prior knowledge of the clinical status. Discordant cases were re-analysed together using a double-headed microscope (Olympus BX46; Olympus Corporation, Center Valley, PA, USA). The p-mTOR expression was semiquantitatively assessed at ×200 magnification, considering the cytoplasmic staining of the tumour and adjacent, non-tumour urothelial cells. The following grading system was used: Negative (−), expression in <10% of cells; and positive (+) expression in ≥10% of cells. CD31 and D2-40 immunohistochemical positive reactions were assessed as previously described, in order to quantify overall BVD and LVD (peritumoural and intratumoural), respectively (37).

### Statistical analysis

Data were analysed using SPSS software for Windows, version 20.0 (IBM, Portsmouth, UK). Associations between p-mTOR expression and the clinicopathological parameters were examined for statistical significance using Pearson’s χ^2^ test or Fisher’s exact test (when n<5). For BVD and LVD analysis, data are expressed as the median, and this value was used as a cut-off point for statistical analysis. Five-year DFS and OS rates were evaluated using Kaplan-Meier curves, and differences were analysed by log-Rank or Breslow tests. P<0.05 was considered to indicate a statistically significant difference.

## Results

### Prognostic significance of the clinicopathological parameters

The 5-year DFS and OS rates were significantly lower in patients with tumours invading beyond the muscular layer, with grade III tumours, with occurrence of lymphovascular invasion or with the presence of regional metastases (Table II). High vascular density did not have an impact on the pathological outcome. However, high LVD was predominant in pT3/pT4 (81%, 33/41, P=0.006), grade III (85%, 35/41, P=0.034) or MI (37/41, 90%, P=0.033) tumours (data not shown).

### Immunoexpression pattern of p-mTOR

**Figure 1:**
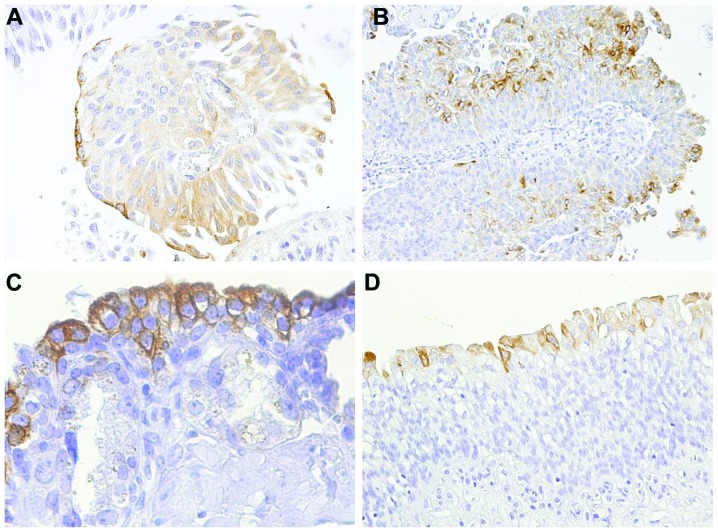
Immunohistochemical positive reactions for p-mTOR, showing different expression patterns in urothelial cells. Non-muscle invasive papillary tumours expressing cytoplasmic p-mTOR in (A) near homogeneous (magnification, ×200) and (B) and heterogeneous (magnification, ×100) patterns. The luminal and intermediate cell layers were more intensely stained than the basal cell layer. (C) Normal (magnification, ×400) and (D) hyperplasic (magnification, ×200) urothelium exhibiting cytoplasmic p-mTOR immunoexpression restricted to the superficial layers. p-mTOR, phosphorylated mammalian target of rapamycin.

A total of 76 UBC samples with representative tumour and non-tumour (normal or hyperplasic urothelium) sections were evaluated for p-mTOR immunoexpression. Of these samples, 20% (15/76) were scored as positive. Regarding NMI papillary lesions, p-mTOR expression was observed to be frequently evenly distributed across the several layers of urothelial cells, with a more intense staining noted in the superficial layers (Fig. 1A). This superficial increase in p-mTOR expression was more evident in some NMI cases, (Fig. 1B). MI positive cases were rare, and p-mTOR was only expressed in a few spots of cells. In cases where non-tumour urothelium, with apparent normal histology (Fig. 1C) or hyperplasic samples (Fig. 1D) were scored as positive (36%, 27/76), p-mTOR expression was completely restricted to the superficial cell layers, predominantly to the umbrella cells.

### Clinical and prognostic significance of p-mTOR immunoexpression

**Figure 2:**
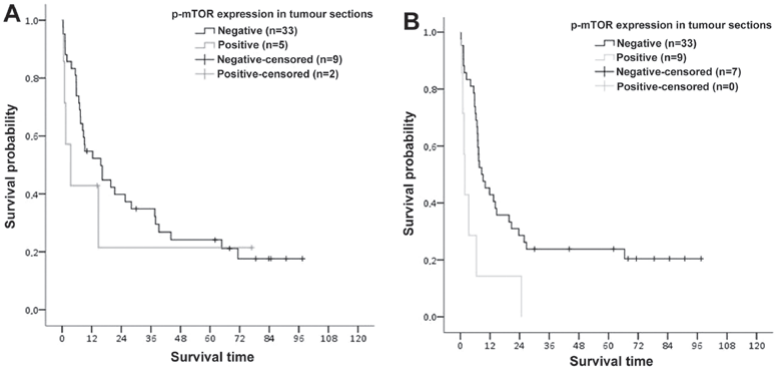
Kaplan-Meier survival curves demonstrating (A) 5-year overall survival (P>0.05) and (B) 5-year disease-free survival (P=0.004) based on p-mTOR immunoexpression status in pT3/pT4 urothelial bladder. P-value presents the differences between the p-mTOR-negative and -positive cases.

The expression of p-mTOR decreased with increasing stage: 40% (6/15) of pT1 and pTis tumours were positively stained, while only 14.3% (7/49) of pT3/pT4 tumours expressed p-mTOR (P=0.087) (Table III). Similar correlations were found when considering the morphological type of lesion (P=0.075) (Table III). When comparing the positive tumour and non-tumour sections, concordance in the expression of p-mTOR was lost with enhanced tumour aggressiveness: 17 pT3/pT4 cases presented positive normal-like mucosal regions adjacent to the tumour sections, but p-mTOR expression was only observed in 6 (35.3%) of these cases (P=0.005, data not shown). Occurrence of angiogenesis and lymphangiogenesis did not correlate with overall expression of p-mTOR. In the group of low blood vessel density count, 65% (26/40) of the cases did not express p-mTOR neither in the tumour nor non-tumour sections (P=0.003, data not shown). No significant associations were observed regarding p-mTOR status and survival rates. However, when selecting the group of patients with pT3/pT4 tumours, those with negative expression had a median 5-year OS of 15.7 months (95% CI, 6.757–24.643), which was reduced to 3.5 months (95% CI, 1.000–8.514) if the tumours were p-mTOR positive, although the differences were not statistically significant (Fig. 2A). Accordingly, the 5-year DFS was reduced from 8.7 months (95% CI, 3.974–13.359) in the negative cases to 1.8 months (95% CI, 1.030–2.570) in the positive cases (P=0.004, Fig. 2B).

## Discussion

The interplay between both mTOR complexes and the PI3K/Akt signalling pathway is support for the consistent upregulation of the mTOR network in numerous cancers. Activating mutations in the *mTOR* gene have been identified in a small number of malignancies, although these have not been clearly associated with tumour development (38). Conversely, upstream components of the mTOR pathway are frequently altered in human tumours (8,15), and UBC is not an exception, with reported mutations of phosphatidylinositol-4,5-bisphosphate 3-kinase, catalytic subunit alpha, *AKT1* and tuberous sclerosis protein 1, hamartin, and loss of heterozygosity, homozygous deletion and inactivating mutations of phosphatase and tensin homologue deleted on chromosome 10 (39,40). These observations strongly suggest that mTOR signalling may be activated in bladder tumours. In accordance with this hypothesis, mTOR inhibition through rapamycin or rapamycin analogues has been shown to reduce proliferation in *in vitro* and *in vivo* UBC models, with corresponding diminished levels of p-S6 (31,32,41). Notably, treatment with mTOR inhibitors has been observed to enhance the therapeutic efficacy of cisplatin and gemcitabine in bladder cancer cell lines (32,42,43), and impair tumour progression when administered intravesically in a bladder cancer mouse model (44). In a phase II study of everolimus treatment in patients with locally advanced or metastatic UBC, clinical activity was demonstrated, and the profile of plasma angiogenesis-related proteins suggested that everolimus exhibits antiangiogenic properties that have a significant function in disease control (45). Despite these promising results, little is known regarding the prevalence and clinical relevance of p-mTOR expression in UBC tissue. A better understanding on this subject may be important to appropriately identify patients with UBC that can achieve benefits from molecularly targeted therapies.

Phosphorylation of mTOR at Ser2448 is often used as an indicator of mTOR activity (15,46). In three studies using the same p-mTOR antibody (with slight differences in the protocols and quantification methods), the percentage of bladder tumour samples with activated mTOR ranged from 32 to 88% (31,32,47). While some authors identified p-mTOR upregulation as an important prognostic factor (31,48), others have found an overall downregulation of the mTOR pathway in UBC (49). Comprehensive immunohistochemical and molecular approaches, encompassing several mTOR upstream and downstream factors, are better suited for investigating the potential impact of this pathway in patients with UBC; however, inconsistent results have been described. Reports on p-Akt (47,48) and p-S6K/p-S6 (31,47,48) upregulation in tumour versus non-tumour urothelium contradict those reporting p-Akt (49) and p-S6 (49,50) downregulation. A few studies have demonstrated positive associations between mTOR pathway activation and the clinicopathological parameters of bladder tumours (48,50), while others have failed to do so (32) or have reported inverse associations (49). It can be argued that heterogeneity among patient selection criteria, and relative proportions of differently staged and graded tumours, immunohistochemical protocols or evaluation of staining methods may significantly contribute to the conflicting results that have been described. However, the unique biological features that define bladder tumourigenesis and tumour progression, together with the intrinsic complexity of the PI3K/Akt/mTOR pathway, are likely the predominant factors in this disease.

In the present study, only p-mTOR expression was evaluated in a cohort of 76 UBC tumours, which although may constitute a limitation, was analysed together with markers of blood and lymphatic endothelium. Only 20% of the tumour samples were scored positive for p-mTOR expression; the adjacent non-tumour urothelium (apparently normal or hyperplasic) was immunostained in 36% of the tissue sections, although only the superficial layers, including umbrella cells, were stained. In the malignant urothelium of NMI lesions, an evenly distributed pattern of expression was frequently observed, but the stronger intensity of staining in the superficial layers was maintained. p-mTOR expression decreased with increasing stage, and MI tumours were predominantly negative. In cases of positive MI tumours, only small clusters of cells were stained. Interestingly, normal-like mucosa of MI lesions preserved p-mTOR expression in a significant proportion of cases that had lost it in the tumour sections. No significant association was found between p-mTOR positivity and neovascularization. When tumour and non-tumour sections were simultaneously negative, the occurrence of angiogenesis was observed to be compromised. In the group of pT3/pT4 tumours, p-mTOR expression was associated with a worse survival rate, although the differences were only significant for 5-year DFS.

The pattern of p-mTOR immunoexpression that was observed in the UBC series of the present study has been similarly described in previous studies (32,48). It may be speculated that the restriction of p-mTOR expression to the superficial layers of the normal-like urothelium reflects the biological plasticity inherent to the epithelial cells, namely the umbrella cells. These cells exhibit unique structural and biochemical features that enable them to form an effective permeable barrier while supporting mechanical deformation due to bladder filling (51,52). It is likely that constitutive expression of mTOR is necessary for normal metabolic activities. It has been previously described that mTORC1, besides being a master regulator of cell growth and proliferation in non-tumour and tumour conditions, additionally controls specific aspects of cellular metabolism through the induction of metabolic gene expression (53–55). Consistent with the results of the present study, NMI tumours may extend and upregulate mTOR expression up to the basal urothelial layer, which is concordant with an enhanced malignant potential that will guide growth and progression of the primary tumour. Fahmy *et al* (56) have recently reported that activation of the mTOR pathway may be used as a predictor of recurrence among patients with high-risk NMI (56). It was shown by Pinto-Leite *et al* (43) that the effect of everolimus in bladder cancer cell lines, alone or in combination with gemcitabine treatment, produced a significant antiproliferative effect for everolimus in an NMI cell line (5637), while an MI cell line (T24) demonstrated marked resistance. These results, together with the results from the present study, suggest that interfering with the mTOR pathway may represent an appealing approach for therapeutic intervention in patients with NMI tumours.

In the present group of MI tumours, two p-mTOR phenotypes were observed. Firstly, positive pT3/pT4 tumours were associated with a worse prognosis, which is in accordance with previous data that have reported upregulation of the mTOR pathway as an important prognostic factor (31,48). Secondly, p-mTOR positivity was rare and restricted to cell spots. In the majority of MI tumour sections, immunoexpression was lost in a *de novo* fashion, as supported by the maintenance of p-mTOR expression in the normal-like adjacent mucosa. It is hypothesised that unknown biological determinants are functioning in the promotion of this unique malignant scenario. Schultz *et al* (49) reported the apparent downregulation of the mTOR pathway, as demonstrated by the low expression levels of p-Akt and p-S6 in invasive UBC, as compared with benign urothelium. It was hypothesised that the downregulation of p-S6 in MI-UBC may be related to the hypoxia-inducible factor-activating hypoxia-resistant microenvironment. Müller *et al* (57) demonstrated that when comparing between normal and prostate tumour tissues, p-mTOR expression was reduced in the tumour, correlating with adverse clinicopathological features. These results, together with the data of the present study, may reflect the occurrence of alternative mTOR signalling mechanisms that underlie the classical PI3K/Akt activation pathway. Additional studies with a larger and more comprehensive UBC series and panels of mTOR upstream and downstream effectors, together with reproducible immunohistochemical and molecular approaches and *in vivo* and *in vitro* bladder tumour models, are required to clarify the mechanism of the mTOR pathway in human UBC, in order to expedite the research on novel therapeutic approaches.

## Figures and Tables

**Table I:** Clinicopathological parameters.

Parameter	n
Gender	
Male	63
Female	13
Age, years	
Median	71
Range	41–83
Tumour stage	
pT1 and pTis	15
pT2	12
pT3 and pT4	49
Grade	
II	19
III	57
Morphological type of lesion	
Non-muscle invasive papillary	11
*In situ*	4
Muscle-invasive	61
Lymphovascular invasion	
Yes	37
No	39
Loco-regional dissemination	
Yes	22
No	54
Recurrence	
Yes	57
No	19
Clinical outcome	
Succumbed to bladder cancer	53
Alive, lost to follow-up, or dead due to other causes	23

**Table II:** Correlation between 5-year disease-free survival and overall survival rates, and clinicopathological variables.

Parameter	n	5-year DFS rate (%)	P-value^a^	5-year OS rate (%)	P-value^a^
Gender					
Male	63	22.0	0.608	31.3	0.780
Female	13	34.6		34.2	
Age, years					
≤71	40	25.6	0.288	33.5	0.317
>71	36	22.4		30.3	
Stage					
pT1 and pTis	15	36.1	0.011	46.5	0.005
pT2	12	27.8		45.8	
pT3 and pT4	49	20.4		23.7	
Grade					
II	19	45.5	0.007	61.2	0.001
III	57	17.2		22.3	
Morphological type of lesion					
Non-muscle invasive papillary	11	30.7	0.059	48.5	0.048
*In situ*	4	50.0		50.0	
Muscle-invasive	61	21.5		28.1	
Lymphovascular invasion					
Negative	39	30.2	0.040	42.9	0.004
Positive	37	18.9		21.0	
Loco-regional metastasis					
Negative	54	28.8	0.043	41.1	0.001
Positive	22	13.6		10.0	

a Obtained using the log-rank or Breslow test.

DFS, disease-free survival; OS, overall survival.

**Table III:** Correlation between p-mTOR expression status in tumour sections and clinicopathological variables.

		p-mTOR expression (%)		
			
Parameter	n	Negative	Positive	P-value^a^
Gender				
Male	63	48 (76.2)	15 (23.8)	0.060
Female	13	13 (100)	0 (0.0)	
Age, years				
≤71	40	31 (77.5)	9 (22.5)	0.534
>71	36	30 (83.3)	6 (16.7)	
Stage				
pT1 and pTis	15	9 (60.0)	6 (40.0)	0.087
pT2	12	10 (83.3)	2 (16.7)	
pT3 and pT4	49	42 (85.7)	7 (14.3)	
Grade				
II	19	14 (73.7)	5 (26.3)	0.507
III	57	47 (82.5)	10 (17.5)	
Morphological type of lesion				
Non-muscle invasive papillary	11	7 (63.6)	4 (36.4)	0.075
*In situ*	4	2 (50.0)	2 (50.0)	
Muscle-invasive	61	52 (85.2)	9 (14.8)	
Lymphovascular invasion				
Negative	39	29 (74.4)	10 (25.6)	0.252
Positive	37	32 (86.5)	5 (13.5)	
Loco-regional metastasis				
Negative	54	42 (77.8)	12 (22.2)	0.532
Positive	22	19 (86.4)	3 (13.6)	
Median BVD (CD31 stain)				
<17.6	40	33 (82.5)	7 (17.5)	0.774
≥17.6	36	28 (77.8)	8 (22.2)	
Median LVD (D2-40 stain)				
<8.8	35	26 (74.3)	9 (25.7)	0.259
≥8.8	41	35 (85.4)	6 (14.6)	

a Obtained using the χ^2^ or Fisher exact test.

BVD, blood vessel density; LVD, lymphatic vessels density.
